# Unlocking Chemotherapy Success: The Role of Diffusion Tensor Imaging in Breast Cancer Treatment

**DOI:** 10.3390/diagnostics14232650

**Published:** 2024-11-24

**Authors:** Anca Ileana Ciurea, Ioana Bene, Paul Cheregi, Thea Brad, Cristiana Augusta Ciortea, Georgeta Mihaela Rusu, Larisa Dorina Ciule, Andrada-Larisa Deac, Manuela Lavinia Lenghel

**Affiliations:** 1 Department of Radiology, Faculty of Medicine, “Iuliu Hațieganu” University of Medicine and Pharmacy, 400012 Cluj-Napoca, Romania; ancaciurea@hotmail.com (A.I.C.); mihageorgeta@yahoo.com (G.M.R.); lenghel.manuela@gmail.com (M.L.L.); 2 Department of Radiology, Emergency County Hospital, 400006 Cluj-Napoca, Romania; cristianaciortea@yahoo.com; 3 Faculty of Medicine, “Iuliu Hațieganu” University of Medicine and Pharmacy, 400012 Cluj-Napoca, Romania; cheregip@yahoo.com (P.C.); theadbrad@yahoo.com (T.B.); 4 Department of Oncology, Emergency County Hospital, 400006 Cluj-Napoca, Romania; larisa_ciule@yahoo.com (L.D.C.); andrada_deac@yahoo.com (A.-L.D.); 5 Department of Pharmacology, Toxicology and Clinical Pharmacology, Faculty of Medicine, “Iuliu Hațieganu” University of Medicine and Pharmacy, 400012 Cluj-Napoca, Romania

**Keywords:** diffusion tensor imaging, neoadjuvant chemotherapy, breast cancer, magnetic resonance imaging

## Abstract

**Background:** This study investigates the role of Diffusion Tensor Imaging (DTI) in predicting the response to neoadjuvant chemotherapy (NAC) in patients with locally advanced breast cancer. **Methods:** A Diffusion Tensor Imaging magnetic resonance imaging (DTI MRI) sequence, evaluating water diffusion along tissue structures, was performed before and after two chemotherapy cycles. This study included 23 patients with 27 malignant masses, comparing changes in DTI parameters with Residual Cancer Burden (RCB) scores. **Results**: We found a significant correlation between changes in specific DTI parameters (e.g., λ2, FA, RA) and pathological response, suggesting that DTI could serve as a sensitive marker for early chemotherapy response. However, differences in sensitivity were observed between DTI sequences with 6 and 12 directions, indicating that 12-direction DTI may provide better diagnostic accuracy. The percentage change in DTI parameters, particularly FA, demonstrated a strong ability to predict pathological complete response (pCR) with high sensitivity. **Conclusions:** As a non-invasive tool, DTI has the potential to assess chemotherapy efficacy, although larger studies with standardized protocols are necessary to validate its clinical utility.

## 1. Introduction

Breast cancer was the leading cause of neoplastic morbidity in 2020, with a worldwide incidence of approximately 2.3 million cases and a mortality of approximately 700,000 deaths [1]. There is a wide variety of risk factors that are related to prolonged exposure to estrogen hormones (early menarche, late menopause, nulliparity, the use of contraceptives, hormone replacement therapy) or to hereditary cancer history and lifestyle characteristics [2].

Neoadjuvant chemotherapy (NAC) is administered with the aim of reducing the tumor size; thus, an initially inoperable tumor or one with an indication for mastectomy ends up being operable through conservative surgical intervention [3]. Another reason justifying neoadjuvant therapy is that it can be used to guide subsequent systemic treatments based on the response observed to preoperative administration [4]. The choice of treatment is made according to the subtype of breast cancer, the stage of the disease and the clinical/biological status of the patient.

Imaging techniques commonly used to assess the response to neoadjuvant therapy are mammography and ultrasound [5]. Based on mammography, changes in tumor size and density can be evaluated; the accuracy of the measurements is influenced by the level of demarcation of the tumor compared to the surrounding breast tissue so that for ill-defined tumors, ultrasound or magnetic resonance imaging should be used [6]. Ultrasound assesses tumor sizes after treatment with greater accuracy than mammography [7]. Magnetic resonance imaging (MRI), through its high resolution of soft-tissue visualization, represents the imaging method with the highest accuracy in evaluating the response to neoadjuvant therapy [8]. Clinical assessment of the response can be reported using RECIST 1.1 criteria, which assess changes in maximum lesion diameter before and after treatment.

The ACRIN 6657 study demonstrated that using functional tumor volume provides greater sensitivity in estimating treatment response compared to measuring maximum tumor diameter. Functional tumor volume is influenced not only by tumor morphology but also by microvascular characteristics. This volume was automatically calculated during the post-processing of MRI scans, with only the voxels showing post-contrast signal enhancement above a predefined threshold being included in the calculation [9]. However, there is currently no standardized procedure for evaluating and reporting the effectiveness of neoadjuvant treatment [10]. One pathology system used for assessing response to neoadjuvant therapy is the Residual Cancer Burden (RCB) system, which includes six parameters that can be easily determined during the pathological examination [10]. If in the past the pathological response to treatment was dichotomously expressed as pathological complete response or residual disease present, with the introduction of the RCB system, patients without pathological complete response are classified into several classes, depending on the extent of residual disease [11]. The standardized assessment approach, the reproducibility of measurements between different pathologists, the use of parameters that do not involve additional costs, and the prognostic information provided in cases where a pathological complete response is not obtained are among the most important advantages to justify the use of the RCB system [11]. The breast tissue, through its structure characterized by the presence of a tree-like duct system, presents a unique model for achieving the diffusion of water molecules. This movement can be made freely along the ducts, so in a direction parallel to the walls of the ducts, and is restricted in the directions perpendicular to the ducts. However, if the ducts are obstructed by a cancer, the mobility of water molecules will be reduced in all directions [12]. The magnetic resonance sequence known as Diffusion Tensor Imaging (DTI) evaluates the diffusion of water in at least six directions, an aspect that allows for establishing the main direction of water mobility.

Despite advancements in breast cancer imaging, traditional techniques like mammography, ultrasound, and even standard MRI have limitations in accurately assessing response to neoadjuvant chemotherapy (NAC), especially in cases of diffuse or poorly defined tumors. The introduction of DTI brings a novel approach by enabling the measurement of water molecule diffusion within breast tissue. DTI can reveal structural changes that correspond to cellular density and tissue architecture, providing unique insight into tumor microenvironment alterations that standard imaging may overlook. This is particularly relevant in breast tissue with its ductal structures, where water diffusion patterns can reflect the presence of obstructions like tumors. By evaluating water mobility in multiple directions, DTI has the potential to distinguish between viable and non-viable tumor tissue earlier in the treatment process. Knowing the existence of numerous adverse effects related to chemotherapy and that some patients with locally advanced breast cancer will not benefit from neoadjuvant therapy, the development of a method for early identification of this category of patients will allow for changing the therapeutic approach in their case.

We utilized 6- and 12-direction DTI protocols to compare their performance and determine if one offers superior and more valuable information. The 6-direction protocol is advantageous for its shorter scan times, lower costs, and suitability for basic metrics like fractional anisotropy (FA), making it ideal for clinical use or for patients with limited scan tolerance. By contrast, the 12-direction protocol excels in providing more accurate tensor estimation, improved noise compensation, and enhanced fiber tracking, particularly in regions with complex fiber crossings, making it indispensable for advanced research and detailed white matter analysis. Based on these considerations, we hypothesized that 12-direction DTI could yield more precise and informative results.

This study aims to evaluate the ability of DTI sequences performed after two cycles of chemotherapy to predict response to neoadjuvant treatment.

## 2. Materials and Methods

This prospective study was approved by the Institutional Review Board (Number/Date: 280/11 August 2020), and informed consent was obtained from each patient before performing MRI. The informed consent included information related to the purpose and duration of the study; risks; benefits; responsibility of the participant; the rights of the participant to withdraw from the study; confidentiality; and the procedure, including the means of administration of the contrast agent and contraindications.

### 2.1. Patients’ Selection

This prospective monocentric study was carried out in the radiology department of the Clinical County Emergency Hospital in Cluj-Napoca, between December 2020 and November 2023. Patients with locally advanced breast cancer, proved by percutaneous biopsy and complete pathological examination, with indication for neoadjuvant therapy were included in the study. Neoadjuvant treatment was performed according to standard protocols. We excluded from the study patients with metastatic cancer, those who did not require neoadjuvant chemotherapy (directly following surgical treatment), those whose follow-up was not completed or had incomplete histopathology results, patients with incomplete or artifactual examination, or those who did not perform the two required MRI examinations.

Two magnetic resonance examinations were performed on a 1.5 Tesla MRI machine (Signa™ Explorer General Electric, GE Healthcare, Fairfield, Waukesha, WI, USA) with the same acquisition protocol for each patient included in this study; the first MRI examination was performed before starting neoadjuvant treatment (MRI 1), and the second was performed after two cycles of chemotherapy (MRI 2). The acquisition protocol included morphological non-enhanced sequences T1, T2, DWI and DTI diffusion sequences with 6 and 12 directions, respectively, and DISCO T1 FATSAT sequences performed dynamically after contrast administration (DCE-MRI) of 0.1 mmol/kg bodyweight of Gadovist® (Bayer AG, Leverkusen, Germany). DTI diffusion sequences were acquired before contrast administration. T1-weighted imaging highlights differences in tissues based on their longitudinal relaxation times (T1). The contrast in T1-weighted images (T1WI) depends on how quickly excited protons relax back to their equilibrium state, a process influenced by the surrounding tissue environment. This contrast is achieved by carefully setting the repetition time (TR) and echo time (TE). For T1-weighted images, a short TR (400–600 ms) and a short TE (10–20 ms) are typically used. T2-weighted images are a type of MRI that emphasizes differences in the T2 relaxation times of tissues. T2 relaxation involves the gradual decay of transverse magnetization (Mxy) after an external radiofrequency (RF) pulse. These images are widely used in clinical imaging to reveal details about tissue water content and other structural characteristics. Diffusion-weighted magnetic resonance imaging (DW MRI) generates image contrast based on the random microscopic movement of water protons, which can be significantly affected by various pathological changes. DW MRI uses a T2-weighted pulse sequence enhanced with two additional gradient pulses of equal strength but opposite directions. These pulses are applied between the nuclear spin excitation and data acquisition, making the imaging more sensitive to water molecule motion along the direction of the added gradients. DTI is a type of diffusion-weighted imaging (DWI) that measures the rate and direction of water diffusion in tissues. In isotropic diffusion, water moves equally in all directions, as in free water, whereas in anisotropic diffusion, water movement has directionality, as along aligned structures like nerve fibers. The degree of anisotropy reflects tissue characteristics, allowing DTI to produce clinically valuable images, especially for examining neural pathways [13].

### 2.2. Image Processing and Data Collection

Image processing was performed on the workstation of the magnetic resonance machine (Advantage workstation 4.7 edition from General Electric). In order to be consistent, we followed the same protocol for the ROI placement in all patients. A circular ROI with a defined area of 5.8 mm^2^ was placed at the level of the lesion identified on the DCE-MRI sequence so that it encompassed an area with homogeneous signal and no areas of necrosis. The time–signal intensity curve was generated for the ROI to be placed in the area of the lesion with the highest signal enhancement after contrast administration (the peak area of enhancement). An ROI of the same size was automatically placed by the software on the MRI workstation at the level of the lesion in the DTI sequence, in the slice corresponding to the DCE-MRI sequence. Numerical values were obtained for the following DTI-derived parameters: λ1, λ2, λ3, FA (fraction of anisotropy), and RA (relative anisotropy). In this phase, patients with incomplete or artifactual examinations were excluded from this study. The difference between λ1 and λ3, as well as the percentage change in each DTI parameter between the two examinations, was calculated.

### 2.3. Postoperative Pathological Examination

After the end of the neoadjuvant treatment, the patients underwent surgery, and the resection specimens were examined to evaluate the response to the treatment. Achieving an RCB score of 0 is equivalent to pathologic complete response. Patients in whom the pathological evaluation of the treatment response did not include the Residual Cancer Burden (RCB) score were excluded from this study.

### 2.4. Statistical Processing

Statistical data processing was performed using the Jamovi statistical program (version 2.5; Sydney, Australia) [14]. Data normality was assessed with the Shapiro–Wilk test. The correlation between the percentage change in DTI parameters between examinations and RCB class was evaluated using the Spearman correlation coefficient. For the following comparisons, two subgroups of patients were defined based on the RCB score: the first subgroup included the patients who were assigned to the RCB class 0 (the RCB 0 subgroup), and the second subgroup included the patients assigned to the RCB classes 1, 2, or 3 (the non-RCB subgroup 0). The assessment of the statistical significance of the differences between the two subgroups of the parameters derived from the DTI was carried out by using Student’s test for independent samples and equal variances and the Welch test for variables with a normal distribution. For variables that did not respect normality, the Mann–Whitney U test was used. The ability to use the percentage change in DTI parameters between examinations to differentiate the two subgroups was assessed by using ROC (receiver operating characteristics) curves, calculating AUC (area under the curve) and applying the DeLong statistical significance test. All statistical tests used were two-tailed. Results with a *p* value ≤ 0.05 were considered statistically significant, and results with 0.05 < *p* ≤ 0.1 were considered with a tendency toward statistical significance.

## 3. Results

### Patient Data

A total number of 23 patients with 27 malignant masses were examined by magnetic resonance before (MRI 1) and after two cycles of chemotherapy (MRI 2). The patients were between 39 and 71 years, with a mean age of 54 years. Following the pathological examination performed after surgical intervention, a number of 6 patients (26.08%) obtained an RCB score of 0, and 17 patients (73.92%) obtained an RCB score higher than 0.

**Figure 1:**
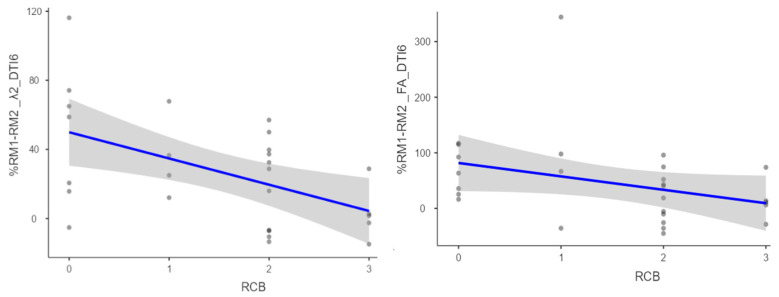
Scatter plots suggesting the association between RCB class and percent change between examinations in 6-direction DTI parameters: λ2 (**left**) and FA (**right**). The blue line indicates the liniar regresion line with a confindence interval of 95% and the dots indicate the data point.

Statistical analysis of the correlation between the percentage change in DTI parameters across examinations and the RCB classification was performed. Statistically significant correlations of an acceptable level were obtained between the RCB class and the percentage change between examinations of the following 6-direction DTI parameters: λ2 (Rho = −0.46 and *p* = 0.014), λ3 (Rho = −0.41 and *p* = 0.033), and FA (Rho = −0.43 and *p* = 0.022) (Figure 1 and Table 1).

**Figure 2:**
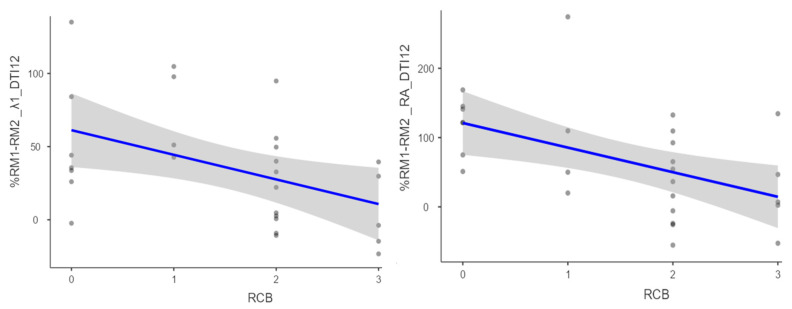
Scatter plots suggesting the association between RCB class and percent change between examinations of the 12-direction DTI parameters: λ1 (**left**) and RA (**right**). The blue line indicates the liniar regresion line with a confindence interval of 95% and the dots indicate the data point.

Statistically significant correlations were obtained between RCB class and the percentage change between examinations of the following 12-direction DTI parameters: λ1 (Rho = −0.48 and *p* = 0.011), λ1–λ3 (Rho = −0.46 and *p* = 0.016), and RA (Rho = −0.55 and *p* = 0.003). Associations with a tendency towards statistical significance were also obtained for the parameters λ2 (Rho = −0.36 and *p* = 0.060) and FA (Rho = −0.36 and *p* = 0.063) (Figure 2 and Table 1).

At pre-treatment magnetic resonance imaging (MRI 1), lower values of all investigated 6-direction DTI parameters were obtained in the subgroup of patients who achieved RCB 0. The differences between the two subgroups were statistically significant for λ1 (*p* = 0.028) and λ1–λ3 (*p* = 0.032), and a trend towards significance was observed for λ2 (*p* = 0.052). After two cycles of chemotherapy (MRI 2), no statistically significant differences were found between subgroups for any of the 6-direction DTI parameters (Table 2).

Similar results were obtained for the 12-direction DTI parameters. At the time of MRI1 in the subgroup of RCB 0 patients, lower values of all investigated parameters were recorded, but statistically significant differences were found only for the RA parameter (*p* = 0.043), and for λ1–λ3 (*p* = 0.086), the difference between subgroups tended to be significant. At MRI2, none of the 12-direction DTI parameters were found to be significantly different between subgroups (Table 3).

**Figure 3:**
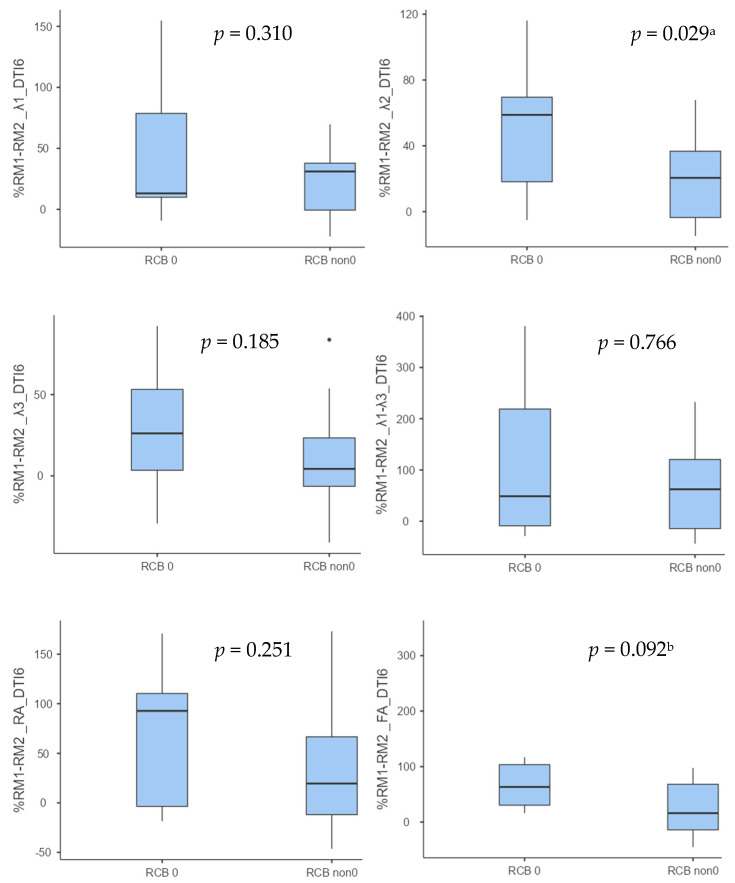
Boxplots showing the percentage change between examinations of 6-way DTI parameters for RCB 0 and non-RCB non subgroups. Statistical analysis of differences between subgroups was performed with Student’s *t* test for independent variables (for normally distributed variables) and the Mann–Whitney U test (for non-normally distributed variables). Statistical significance was obtained for λ2 (*p* = 0.029), and there was a trend towards significance for FA (*p* = 0.092). ^a^ *p* < 0.05, ^b^ *p* < 0.1.

The percentage changes in the 6-direction DTI parameters between the examination performed before NAC and that performed after two cycles of NAC had higher values in the subgroup of patients who achieved RCB 0. These differences were found to be significant only for λ2 (*p* = 0.029), and for FA (*p* = 0.092), the differences approached statistical significance (Table 4 and Figure 3).

**Figure 4:**
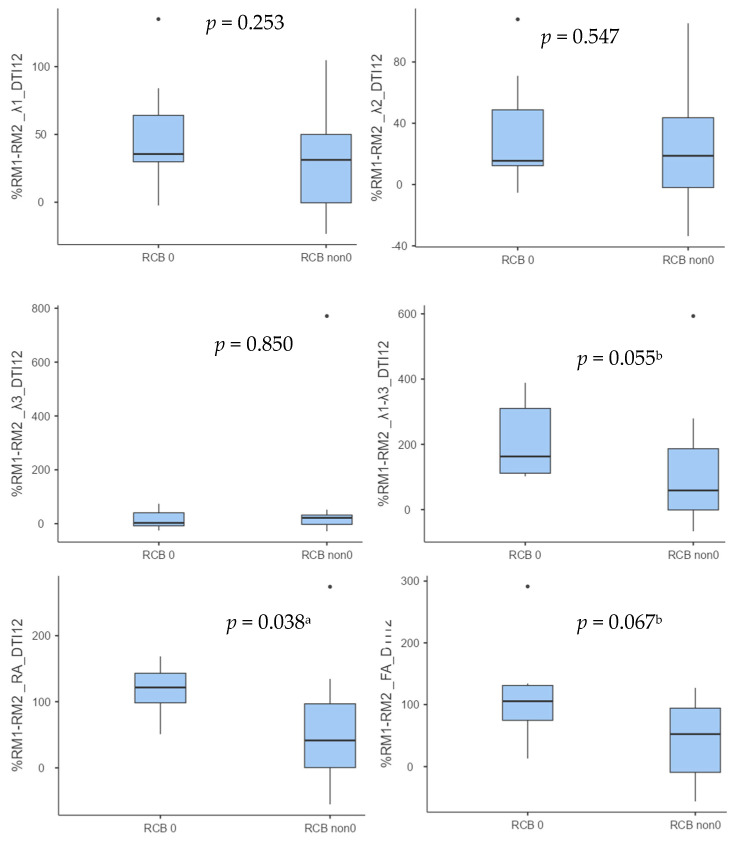
Boxplots showing, for RCB 0 and non-RCB 0 subgroups, the percentage change between examinations in 12-direction DTI parameters. Statistical analysis of differences between subgroups was performed with Student’s *t* test for independent variables (for normally distributed variables) and the Mann–Whitney U test (for non-normally distributed variables). ^a^ *p* < 0.05, ^b^ *p* < 0.1.

Similar results were obtained for the percent change between examinations of the 12-direction DTI parameters. Thus, higher values of all these parameters were recorded in the subgroup of RCB 0 patients. The differences between the subgroups were found to be statistically significant for the parameter RA (*p* = 0.038), and a tendency towards statistical significance was observed for λ1–λ3 (*p* = 0.055) and for FA (*p* = 0.067) (Table 4 and Figure 4).

Statistical significance was obtained for RA (*p* = 0.038), and a trend towards significance for λ1–λ3 (*p* = 0.055) and for FA (*p* = 0.067) was observed. a *p* < 0.05; b *p* < 0.1.

Statistical evaluation was performed for the ability of MRI to identify, based on percentage changes in DTI parameters between MRI1 and MRI2 examinations, patients would achieve a pathological complete response.

**Figure 5:**
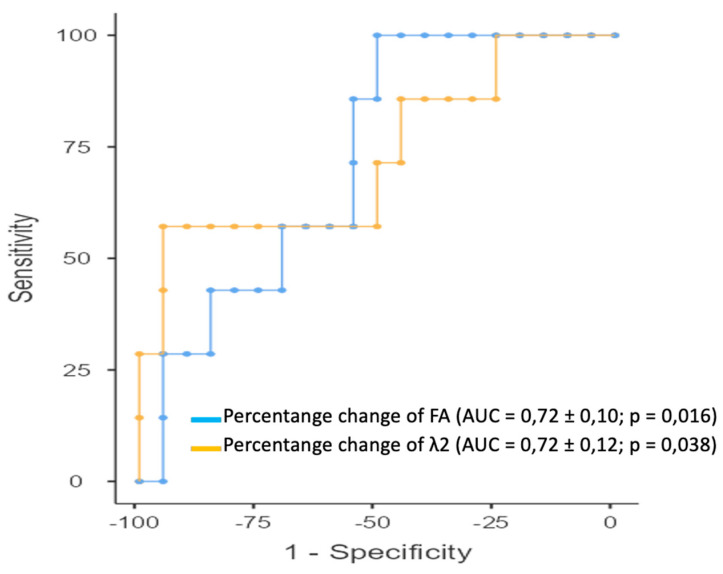
ROC analysis of the use of percentage changes in 6-direction DTI parameters to identify patients who would achieve a pathological complete response following NAC. ROC curves of DTI parameters for which AUC ≥ 0.5 and *p* ≤ 0.05 (*p* value was obtained with the DeLong test) were plotted: FA (AUC = 0.72 ± 0.10; *p* = 0.016) and λ2 (AUC = 0.72 ± 0.12; *p* = 0.038). To compare the two curves, the DeLong test was applied, and it showed that there were no statistically significant differences between the curves (*p* = 0.963).

On ROC curve analysis of the ability of the percentage changes of 6-direction DTI parameters between the two examinations to predict the achievement of RCB score 0, significant associations were obtained for λ2 (AUC = 0.72 ± 0.12; *p* = 0.038) and FA (AUC = 0.72 ± 0.10; *p* = 0.016) (Figure 5). Detailed results of the ROC analysis are shown in Table 5.

**Figure 6:**
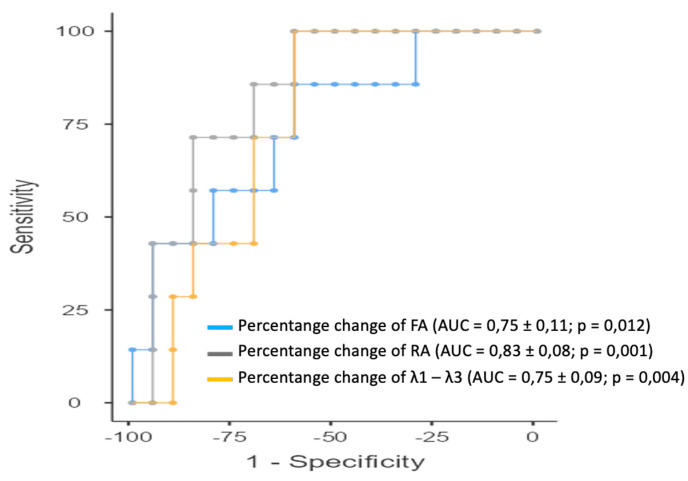
ROC analysis of using percentage changes in 12-direction DTI parameters to identify patients who would achieve a pathologic complete response following NAC. ROC curves of DTI parameters for which AUC ≥ 0.5 and *p* ≤ 0.05 (*p* value was obtained with the DeLong test) were plotted: FA (AUC = 0.75 ± 0.11; *p* = 0.012), RA (AUC = 0.83 ± 0.08; *p* = 0.001) and λ1–λ3 (AUC = 0.75 ± 0.09; *p* = 0.004). To compare the curves between them, the DeLong test was applied, and it revealed no statistically significant differences between the parameters.

Table 6 presents the results of the ROC analysis for the percentage changes in parameters derived from 12-direction DTI between examinations. Statistically significant associations were found between achieving an RCB score of 0 and changes in the following parameters: λ1–λ3 (AUC = 0.75 ± 0.09; *p* = 0.004), FA (AUC = 0.75 ± 0.11; *p* = 0.012), and RA (AUC = 0.83 ± 0.08; *p* = 0.001) (Figure 6).

## 4. Discussion

Neoadjuvant chemotherapy is the standard treatment for locally advanced breast cancer. One of the key benefits of this approach is the potential to enable breast-conserving surgeries. More importantly, it provides the opportunity to assess the in vivo effectiveness of a specific chemotherapy regimen [15].

Diffusion weighted imaging (DWI) is a magnetic resonance sequence that can quantify the diffusion of water molecules at the tissue level, movement that is determined by a physical process called thermal agitation [16]. The degree of water mobility at tissue level is influenced by the level of tissue cellularity and the degree of integrity of cell membranes. Based on the DWI acquisition, the apparent diffusion coefficient (ADC) map is generated, with ADC being a quantitative parameter for diffusion evaluation [16]. Possible clinical applications of DWI include its use as a supplementary tool for breast lesion characterization, a method for breast cancer screening with MRI without contrast material, and a technique for evaluating neoadjuvant systemic treatment [16]. Studies have shown that in breast cancer patients undergoing neoadjuvant therapy, changes in ADC values can be detected earlier than size changes, allowing for an early assessment of treatment response using this parameter [17]. The role of DWI as a method for evaluating response to neoadjuvant treatment has been the subject of several studies. A meta-analysis, which analyzed the results of these studies, concluded that there is large heterogeneity in the level of accuracy of using DWI as a predictor of pathological response between the different primary studies included in the analysis. At the same time, it offered some recommendations on how the following studies should be constructed. The implementation of a standardized approach for acquiring DWI sequences, along with conducting studies on a larger number of subjects and multicenter trials, is recommended [18]. The first multicenter trial conducted with the aim of investigating the role of ADC as a predictive marker of pathological response, in patients undergoing neoadjuvant therapy, was the ACRIN 6698 study, conducted on a group of 242 patients with locally advanced breast cancer. All patients were treated with taxane-based chemotherapy regimens followed by four cycles of anthracyclines and examined by magnetic resonance several times during treatment. A statistically significant association was observed between mid-treatment and post-treatment ADC values and pathologic response, regardless of cancer subtype. If the analysis also took into account the cancer subtype, this association remained significant only for ER+/HER2- mid-treatment and ER-/HER2- post-treatment tumors [19].

The first data on the applicability of DTI in breast pathology showed that DTI can distinguish between breast cancer and normal fibro-glandular tissue based on the FA parameter, which has significantly lower values in the tumors, but it cannot distinguish between benign and malignant lesions [20]. Later, it was proven that DTI can distinguish between a benign and a malignant lesion; the FA values were found to be significantly lower in benign tumors compared to malignant tumors [21].

Further research has evaluated the utility of DTI in monitoring response to neoadjuvant treatment. One such study used a cohort of 34 patients with locally advanced breast cancer undergoing neoadjuvant treatment to investigate the association between DTI-derived quantitative parameters and achievement of pathologic complete response. No significant differences were found in the DTI parameters determined after the third cycle of chemotherapy between the sub-groups of patients with pathological complete response and those without pathological complete response. If the percentage change in these parameters from pretreatment measurements was included in the statistical analysis, significantly greater changes in λ1, λ2, λ3, and ADC were found in the group of patients with pathological complete response, but not for FA. The lack of association of FA with pathological response has been attributed to the greater heterogeneity of FA between participants and the fact that changes in FA may occur later in the course of treatment [22]. Another study, performed on 20 breast cancer patients treated with neoadjuvant chemotherapy, obtained similar results, with significantly higher λ1, λ2, λ3, λ1–λ3, and ADC in responders but with an FA value that, although higher for responders, was statistically insignificant [23]. As a result, DTI is a promising technique for evaluating the response to neoadjuvant treatment, but studies performed on larger groups of patients and with standardized technical acquisition parameters are still needed to be able to validate the value of DTI parameters as biomarkers of pathological response.

This study aimed to evaluate the usefulness of quantitative parameters derived from DTI in determining the response to neoadjuvant chemotherapy in patients with locally advanced breast cancer.

When testing the association between the percentage change in DTI parameters between the two examinations and the RCB class, negative correlations of an acceptable level were obtained for the main eigenvalues, FA and RA. This fact suggests that important percentage changes in these parameters are correlated with reduced or absent residual disease on the post-surgical resection specimen and vice versa. There were differences between 6- or 12-direction DTI sequences in parameters statistically significantly correlated with RCB class. The differences between the two techniques can be explained by an increased sensitivity of the 12-direction DTI sequence in characterizing tissue diffusion compared to the 6-direction one.

Mean tumor-level FA parameter values observed before NAC were similar to those obtained by Partridge et al. [20]; also, these were lower than the FA values from normal breast parenchyma observed in the same study.

The following comparisons assessed the association between DTI parameters and achieving a RCB score of 0, which is equivalent to pathological complete response (pCR). pCR, defined as the absence of residual disease in the breast and at the axillary level (ypT0/is ypN0), is currently the reference method in evaluating the response to NAC. Several meta-analyses based on clinical trials have shown that patients who achieve a pCR following NAC have better survival and lower disease recurrence rates, especially if they had an aggressive, HER2-positive or triple negative breast cancer subtype [24].

During the magnetic resonance examination performed before treatment (MRI 1), the values of all investigated DTI parameters were lower in the subgroup of RCB 0 patients, but the differences were significant for λ1, λ2, λ1–λ3 (6-direction DTI), and λ1–λ3, RA (12-direction DTI). After the second cycle of NAC, none of the DTI parameters were found to be different between the two analyzed subgroups. The results obtained are similar to those obtained by Wilmes et al. [22] in their study on the contribution of diffusion tensors in the assessment of response to NAC. The methodology of the above study assumed the second MRI examination after the third cycle of chemotherapy, and the acquisition of the DTI sequence was performed by applying diffusion gradients only in 6 directions.

When evaluating the association between the percentage change in DTI parameters between examinations and the level of residual disease after treatment, greater increases in these parameters were observed in the subgroup of RCB 0 patients. Among the 6-direction DTI parameters, only for λ2 were the results also significant, and for FA a *p*-value of 0.092 was obtained, suggesting some tendency towards statistical significance. For the 12-direction DTI parameter, FA, the *p*-value approaches the threshold for statistical significance even more closely (*p* = 0.067). Wilmes et al. [22] obtained significantly greater percentage changes in 6-direction DTI parameters for all parameters except for FA (*p* = 0.49). Greater percentage changes in all DTI parameters except FA in the responder subgroup were also obtained by Furman-Haran et al. [23] in their paper. However, clarifications must be made about the methodology of the previously mentioned study. The second magnetic resonance examination was performed after the end of neoadjuvant chemotherapy, 15 diffusion gradients were used to acquire the DTI sequence, and the extent of residual disease in the post-surgical resection specimen was evaluated using the Miller–Payne (M&P) system. Patients who received an M&P score of ≥3 were classified as responders to neoadjuvant treatment. The lack of statistical significance in the previously presented study and the percentage change in FA between pre-treatment and post-neoadjuvant treatment examinations can be attributed to the way in which the treatment response was defined, namely obtaining a M&P score at least equal to three. Another factor that can explain the lack of significance for FA is how the FA values were obtained when processing the acquired images. Wilmes et al. [22] were guided in the positioning of the ROI based on the DWI sequence, the ADC parametric map, and the DCE images (the tumor area in DWI hypersignal with a corresponding hyposignal on the ADC map and showing postcontrast signal enhancement was chosen). Another approach was chosen by Furman-Haran et al., who used, in addition to DCE and T2 images, the λ1 parametric map to identify the lesion (pixels with a λ1 value below 1.73 × 10^−3^ mm^2^/s were included) and guide the positioning of the ROI, which was then automatically transferred by DTI software (Advantage workstation 4.7 edition from General Electric) on the rest of the parametric maps [23]. Baltzer et al. [21] observed that there were differences between the FA values obtained through an ROI encompassing the entire malignant tumor lesion and those obtained through an ROI limited to the area with the most intense signal at the level of the lesion; only the latter differed significantly from the normal breast parenchyma.

Evaluation of the diagnostic performance of magnetic resonance (based on changes in DTI parameters between MRI1 and MRI2 examinations) as a method to identify patients who would achieve a pathological complete response (equivalent to a RCB score) after the end of neoadjuvant chemotherapy revealed contradictory data between the sequences of DTI with 6 and 12 directions for the parameters λ1, λ2, and λ1–λ3 (statistical significance of the area under the curve was obtained for λ2 derived from DTI with 6 directions and for λ1–λ3 derived from DTI with 12 directions). For the FA parameter, the results were similar for the two investigated DTI sequences; thus, for FA from 6-direction DTI, the following were obtained: AUC = 0.72 ± 0.10; *p* = 0.016; Se/Sp = 100/50. For FA from 12-direction DTI, the following were obtained: AUC = 0.75 ± 0.11; *p* = 0.012; Se/Sp = 85.71/60. While the sensitivity values obtained are high, the specificity is modest. This indicates that the percentage change in FA between examinations may primarily serve as a screening method for identifying patients likely to achieve pCR. In other words, it can be confidently stated that if a patient’s FA percentage change between examinations is below the threshold value, they are unlikely to achieve pCR at the conclusion of NAC.

In the study conducted by Furman-Haran et al. [23], which involved 20 patients who underwent pre- and post-treatment imaging using 1.5 T MRI, the researchers specifically focused on comparing DTI metrics with dynamic contrast-enhanced (DCE) MRI in evaluating tumor size and treatment response. They used Pearson’s correlation and other statistical tests to assess the accuracy of DTI parameters in reflecting the tumor’s changes, which were also evaluated by histopathological grading. The findings revealed a strong correlation between DTI and DCE measurements of tumor volume changes, with a Pearson correlation coefficient of 0.82. Moreover, changes in DTI parameters, particularly the volume changes and the diffusion coefficients, significantly correlated with pathological findings and Miller and Payne (M&P) grading, which classifies treatment response. DTI was able to differentiate between chemotherapy responders and non-responders with a receiver operating characteristic (ROC) area under the curve (AUC) of approximately 0.83–0.84, similar to DCE MRI. Notably, the study found that DTI could predict the residual tumor size post-chemotherapy with high accuracy, matching histological findings closely. These results underscore the potential of DTI as a valuable non-invasive tool for assessing breast cancer response to neoadjuvant chemotherapy. It shows promise in offering an early, accurate method for monitoring tumor changes.

This study has a several limitations. One of them is the small number of patients included in this study. The studied group included patients with locally advanced breast cancer, but not all with the same intrinsic subtype, and this aspect was not taken into account in the comparisons made. The validity of the obtained results could also be affected due to the use of different chemotherapy regimens in the patients. The comparison of the obtained results with those existing in the literature was difficult due to the small number of studies published on the studied topic, due to methodological differences between the studies and through the acquisition parameters of the different DTI sequences. Larger patient cohort studies with well-established and identical protocols (standardized DTI acquisition parameters, uniform image post-processing algorithm, statistical processing to account for intrinsic breast cancer subtype and patient regimen) are needed to evaluate the applicability of DTI as a method for evaluating the response to neoadjuvant chemotherapy.

## 5. Conclusions

The percentage change in the FA parameter between examinations can identify, with high sensitivity, patients who will achieve pCR following neoadjuvant chemotherapy. The percentage change between examinations of DTI parameters correlates (negatively) with RCB class. Based only on an examination performed after two cycles of NAC, it is not possible to identify the patients who will achieve RCB 0 following NAC.

## Figures and Tables

**Table 1:** Correlations (Spearman) between percent change between examinations of DTI parameters and RCB class.

Percentage Change Between Examinations—6-Direction DTI	Rho	*p*-Value	Statistical Significance
λ1	−0.24	0.215	Weak or no correlation
λ2	−0.46	0.014 ^a^	Acceptable degree of association
λ3	−0.41	0.033 ^a^	Acceptable degree of association
λ1–λ3	−0.02	0.910	Weak or no correlation
FA	−0.43	0.022 ^a^	Acceptable degree of association
RA	−0.20	0.315	Weak or no correlation
Percentage Change Between Examinations—12-Direction DTI			
λ1	−0.48	0.011 ^a^	Acceptable degree of association
λ2	−0.36	0.060 ^b^	Acceptable degree of association
λ3	−0.25	0.197	Weak or no correlation
λ1–λ3	−0.46	0.016 ^a^	Acceptable degree of association
FA	−0.36	0.063 ^b^	Acceptable degree of association
RA	−0.55	0.003 ^a^	Moderate to good correlation

^a^ *p* < 0.05, statistically significant; ^b^ *p* < 0.1, tendency towards statistical significance; FA, fraction of anisotropy; RA, relative anisotropy.

**Table 2:** Comparison between RCB 0 and RCB non 0 subgroups of 6-direction DTI parameters before (MRI 1) and after two cycles of chemotherapy (MRI 2).

	RCB 0 (*n* = 7)	RCB Non 0 (*n* = 20)	*p*-Value	Cohen’s Kappa
MRI 1 DTI 6				
λ1 (×10^−10^)	11.60 ± 1.13	14.30 ± 2.98	0.028 ^a^	−1.02
λ2 (×10^−10^)	10.00 ± 1.28	12.20 ± 2.65	0.052 ^b^	−0.89
λ3 (×10^−10^)	8.90 ± 1.31	10.30 ± 2.38	0.151	−0.65
λ1–λ3 (×10^−10^)	2.70 ± 0.49	4.01 ± 1.48	0.032 ^a^	−0.99
RA	0.11 ± 0.02	0.13 ± 0.04	0.186	−0.59
FA	0.13 ± 0.03	0.16 ± 0.05	0.170	−0.62
MRI 2 DTI 6				
λ1 (×10^−10^)	12.90 (12.70–20.90)	17.70 (15.40–19.90)	0.455	0.20
λ2 (×10^−10^)	14.80 ± 3.84	14.40 ± 3.96	0.846	0.08
λ3 (×10^−10^)	11.30 ± 3.48	11.30 ± 3.62	0.960	0.02
λ1–λ3 (×10^−10^)	5.67 ± 4.05	6.16 ± 2.83	0.723	−0.15
RA	0.18 ± 0.08	0.16 ± 0.06	0.501	0.29
FA	0.22 ± 0.06	0.20 ± 0.07	0.573	0.25

^a^ *p* < 0.05, statistically significant; ^b^ *p* < 0.1, trend towards statistical significance; *p* values were obtained by applying Student’s test for independent variables; the nonparametric Mann–Whitney U test; mean ± standard deviation for normally distributed data; median (quartile 1–quartile 3) for data that are not normally distributed; FA, fraction of anisotropy; RA, relative anisotropy; Cohen Kappa values < 0 no agreement and ≤0 ≤ 0.20 slight agreement, <0.21 ≤ 0.40 fair agreement, <0.41 ≤ 0.60 moderate agreement, <0.61 ≤ 0.80 substantial agreement, and <0.81 ≤ 1 almost perfect agreement.

**Table 3:** Comparison between RCB 0 and RCB non 0 subgroups of 12-direction DTI parameters before (MRI 1) and after two cycles of chemotherapy (MRI 2).

	RCB 0 (*n* = 7)	RCB Non 0 (*n* = 20)	Valoare *p*	Cohen’s Kappa
MRI 1 DTI 12				
λ1 (×10^−10^)	12.40 ± 1.27	13.90 ± 3.15	0.242	−0.52
λ2 (×10^−10^)	11.30 ± 1.16	12.20 ± 2.59	0.361	−0.40
λ3 (×10^−10^)	10.10 (9.27–11.00)	10.30 (9.75–10.90)	0.803	0.07
λ1–λ3 (×10^−10^)	2.00 (1.85–2.58)	2.80 (2.34–4.28)	0.086 ^b^	0.45
RA	0.08 ± 0.02	0.11 ± 0.03	0.043 ^a^	−0.93
FA	0.10 ± 0.04	0.14 ± 0.05	0.199	−0.58
MRI 2 DTI 12				
λ1 (×10^−10^)	18.60 ± 5.42	17.60 ± 4.97	0.664	0.19
λ2 (×10^−10^)	15.10 ± 4.36	15.10 ± 4.72	0.991	0.01
λ3 (×10^−10^)	11.80 ± 3.64	12.10 ± 3.27	0.826	−0.09
λ1–λ3 (×10^−10^)	6.60 (5.21–8.91)	4.51(4.04–6.03)	0.309	0.45
RA	0.17 ± 0.06	0.15 ± 0.05	0.337	0.42
FA	0.20 ± 0.04	0.18 ± 0.06	0.345	0.42

^a^ *p* < 0.05, statistically significant; ^b^ *p* < 0.1, trend towards statistical significance; *p* values were obtained by applying Student’s test for independent variables; the nonparametric Mann–Whitney U test; mean ± standard deviation for normally distributed data; median (quartile 1–quartile 3) for data that are not normally distributed; FA, fraction of anisotropy; RA, relative anisotropy; Cohen Kappa values < 0 no agreement and ≤0 ≤ 0.20 slight agreement, <0.21 ≤ 0.40 fair agreement, <0.41 ≤ 0.60 moderate agreement, <0.61 ≤ 0.80 substantial agreement, and <0.81 ≤ 1 almost perfect agreement.

**Table 4:** Comparison between RCB 0 and non-RCB 0 subgroups of percentage change between examinations (MRI 1 and MRI 2) of DTI parameters.

Percentage Change Between Examinations 6-Direction DTI	RCB 0 (*n* = 7)	RCB Non 0 (*n* = 20)	Valoare *p*	Cohen’s Kappa
λ1	47.97 ± 60.24	22.27 ± 23.80	0.310	0.56
λ2	49.32 ± 41.54	19.01 ± 24.90	0.029 ^a^	1.01
λ3	28.89 ± 41.25	9.46 ± 29.17	0.185	0.59
λ1–λ3	48.64 (−9.01–218.90)	62.25 (−14.53–120.43)	0.766	0.08
RA	65.45 ± 73.59	31.84 ± 62.25	0.251	0.51
FA	63.39 (30.53–103.63)	16.14 (−14.08–68.33)	0.092 ^b^	0.44
Percentage Change Between Examinations 12-Direction DTI				
λ1	50.87 ± 45.14	30.35 ± 38.17	0.253	−0.36
λ2	34.32 ± 40.20	24.43 ± 35.79	0.547	−0.59
λ3	2.97 (−7.79–40.54)	21.84 (−2.48–32.29)	0.850	0.05
λ1–λ3	162.85 (111.46–310.17)	58.72 (−1.18–186.57)	0.055 ^b^	0.50
RA	117.71 ± 41.21	49.39 ± 78.02	0.038 ^a^	0.96
FA	117.36 ± 86.92	51.19 ± 75.87	0.067 ^b^	0.84

^a^ *p* < 0.05, statistically significant; ^b^ *p* < 0.1, trend towards statistical significance; *p* values were obtained by applying Student’s test for independent variables; the nonparametric Mann–Whitney U test; mean ± standard deviation for normally distributed data; median (quartile 1–quartile 3) for data that are not normally distributed; FA, fraction of anisotropy; RA, relative anisotropy; Cohen Kappa values < 0 no agreement and ≤0 ≤ 0.20 slight agreement, <0.21 ≤ 0.40 fair agreement, <0.41 ≤ 0.60 moderate agreement, <0.61 ≤ 0.80 substantial agreement, and <0.81 ≤ 1 almost perfect agreement.

**Table 5:** ROC analysis of the ability of percentage changes of 6-way DTI parameters between examinations to identify patients who will achieve a pathological complete response.

Percentage Change Between Examinations 6-Direction DTI	AUC	Cut-Off (%)	Se/Sp (%)	*p*-Value
λ1	0.57 ± 0.15	55.00	42.86/95	0.325
λ2	0.72 ± 0.12	58.80	57.17/95	0.038 ^a^
λ3	0.65 ± 0.15	26.14	57.14/80	0.158
λ1–λ3	0.54 ± 0.14	338.66	28.57/100	0.385
FA	0.72 ± 0.10	16.29	100/50	0.016 ^a^
RA	0.64 ± 0.13	92.68	57.14/85	0.142

^a^ *p* < 0.05, statistically significant; AUC ± standard deviation; *p* values were obtained by applying the DeLong test; FA, fraction of anisotropy; RA, relative anisotropy; AUC, area under the curve; Se, sensitivity; Sp, specificity.

**Table 6:** ROC analysis of the ability of percentage changes in 12-direction DTI parameters between examinations to identify patients who would achieve a pathological complete response.

Percentage Change Between Examinations—12-Direction DTI	AUC	Cut-Off (%)	Se/Sp (%)	*p*-Value
λ1	0.62 ± 0.12	26.01	85.71/45	0.157
λ2	0.54 ± 0.13	12.17	85.71/35	0.374
λ3	0.47 ± 0.12	55.26	18.57/95	0.425
λ1–λ3	0.75 ± 0.09	101.92	100/60	0.004 ^a^
FA	0.75 ± 0.11	69.34	85.71/60	0.012 ^a^
RA	0.83 ± 0.08	51.06	100/60	0.001 ^a^

^a^ *p* < 0.05, statistically significant; AUC ± standard deviation; *p* values were obtained by applying the DeLong test; FA, fraction of anisotropy; RA, relative anisotropy; AUC, area under the curve; Se, sensitivity; Sp, specificity.

## Data Availability

The original contributions presented in the study are included in the article, further inquiries can be directed to the corresponding author.
